# Anatomical Study of the Nerve Supply of the Dromedary Camel (*Camelus dromedarius*) in the Distal Hindlimb with a Special Reference to the Cutaneous Innervation

**DOI:** 10.3390/vetsci10040305

**Published:** 2023-04-21

**Authors:** Gamal M. Allouch, Fahad A. Alshanbari

**Affiliations:** Department of Veterinary Medicine, College of Agriculture and Veterinary Medicine, Qassim University, Buraydah 51452, Saudi Arabia

**Keywords:** animals, nerve, camels, distal hind limb, anatomical study

## Abstract

**Simple Summary:**

The nerve supply of the distal part of the hindlimb is very important for the motor and sensory function of the hindlimb. The dromedary camel is historically and currently a very important species for transportation, riding, and racing in many countries. Therefore, understanding the structural components of its limbs is highly important for clinical and surgical purposes. The nerve supply of the distal part of the hindlimb has been discussed in a few domestic species; however, little is known about the nerve supply in the dromedary camel. This study aimed to show the anatomical structure of the nerve supply of the distal part of the hindlimb. Dromedary hindlimbs were collected from a slaughterhouse. Then, they were fixed using 10% formalin. Subsequently, dissection was performed to show the group of nerves that supply the hindlimb’s distal portion. The result shows the branches of the superficial fibular nerve and tibial nerve. It is very important to understand the nerves that supply the distal part of the hindlimb for anesthesia of the skin, tendons and joints.

**Abstract:**

This study aimed to describe the anatomy of the nerve supply of the hindlimb’s distal portion in a dromedary camel’s foot. In our study, we used ten adult slaughtered dromedary camels (twenty distal hindlimbs) of different sexes and ages (4–6 years). The hindlimbs were preserved using 10% formalin for about one week. The distal part of the hindlimb of the camels was dissected with extreme precision to show the group of nerves responsible for the nervous supply to the distal part of the hindlimb in dromedary camels. This study shows the numerous branches of the superficial fibular nerve along its extension to the dorsal surface metatarsus and the abaxial aspect of the third digit. The results show that the tibial nerve possesses many branches along its extension to the plantar surface skin of the metatarsus. Additionally, it provides the axial and abaxial plantar surfaces of the fourth digit and the interdigital surfaces as well as its branches to supply the plantar-abaxial and plantar-axial of the third digit. The present study shows the anatomical nerve supply of the hindlimb’s distal portion that is essential for anesthesia and surgery in this region.

## 1. Introduction

The origin and distribution of the sciatic and femoral nerves have been studied in bovines [1,2], sheep and goats [3], horses [4], and cats [5]. However, detailed descriptions of which nerves feed the pes of the hindlimb are still limited, with authors rarely addressing the distribution of nerves distally to the stifle joint in various domestic animals [6,7,8,9,10,11]. The nerve supply of the distal portion of the hindlimb in camels is very important for the motor and sensory function of the skin and structures of the hindlimb. Branches of the nerve supply in the lower part of the hindlimb have been described in detail in domestic animals, and more particularly in the dromedary camel [12,13,14,15,16,17]. The terminal branches of the ischiatic nerve branches represented by the fibular and tibial nerves contribute to the nerve supply of the distal region of the hindlimb [14]. The common fibular nerve divided into superficial and deep fibular nerves occurs in the middle third of the metatarsus [12,15]. In bovines, it has been found that the superficial fibular nerve splits into the common dorsal digit nerves II, III and IV, while the dorsal metatarsal nerve III arises from the common peroneal nerve [16]. The tibial nerve splits into medial and lateral plantar nerves, confirming the pattern that is described in other animals [12,13,14,15,16,17,18]. The medial plantar nerve continues as common plantar digital nerves II and III, which also continues as plantar proper axial nerves of digits III and IV [14].

Knowledge of the position and distribution of nerves in the distal parts of limbs is of great importance, especially for treating tendonitis, osteoarthritis and sesamoiditis in dogs [19]. The objective of this study was to provide some details about the nerve supply in the distal hindlimb of the camel.

## 2. Materials and Methods

For this work, we used twenty distal hindlimbs of ten freshly slaughtered adult dromedary camels of different sexes and ages (4–6 years). The specimens were obtained from a typical Buraydah slaughterhouse, Qassim Region, KSA. The hindlimb samples were fixed in 10% formalin solution for about one week. Then, samples were washed with distal water, and the skin was removed to be dissected. In order to remove the tissue around the nerves of the distal hindlimb, the nerves were gently massaged with gauze pieces bathed in 1% glacial acetic acid [20,21]. The details of the location and course of supplied nerves to the distal part of hindlimb were described carefully.

## 3. Results

The distal part of the hindlimb in camels receives its nerve supply from the terminal branches of the ischiatic nerve, including the common fibular or (peroneal) and tibial nerves. 

### 3.1. Superficial Fibular Nerve (N. fibularis superficialis)

**Figure 1:**
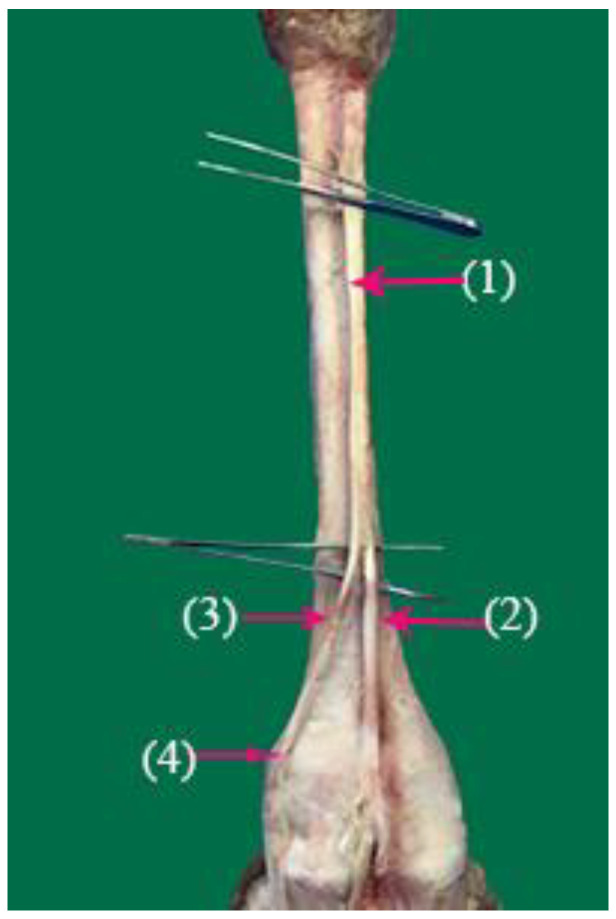
Nerve supply of the dorsal surface of the distal hindlimb in camels: (1) superficial fibular nerve; (2) common dorsal digital nerve of the third digit; (3) common dorsal digital nerve of the fourth digit; (4) abaxial dorsal proper digital IV nerve.

**Figure 2:**
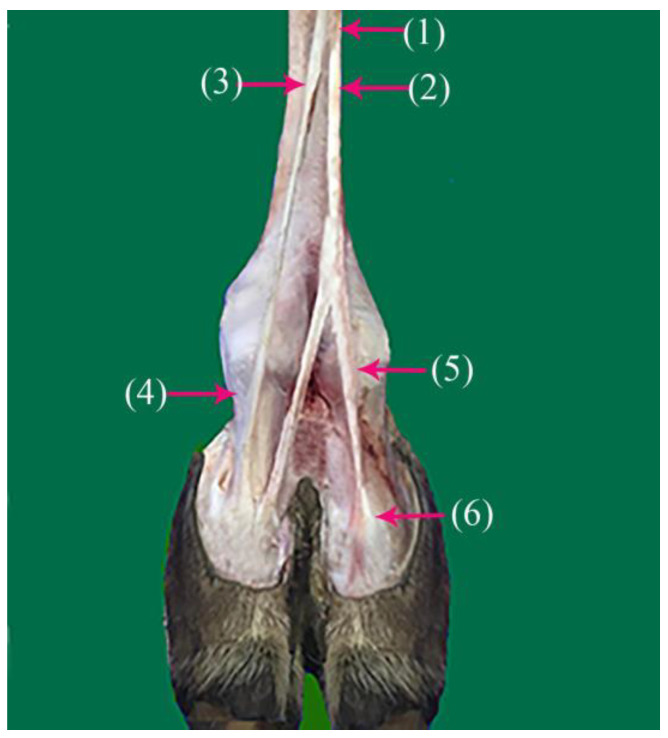
Nerve supply of the dorsal surface of the phalanx of the hindlimb in camels: (1) superficial fibular nerve; (2) common dorsal digital nerve of the third digit; (3) common dorsal digital nerve of the fourth digit; (4) abaxial dorsal proper digital IV nerve; (5) axial dorsal proper digital IV nerve; (6) abaxial dorsal proper digital III nerve.

**Figure 3:**
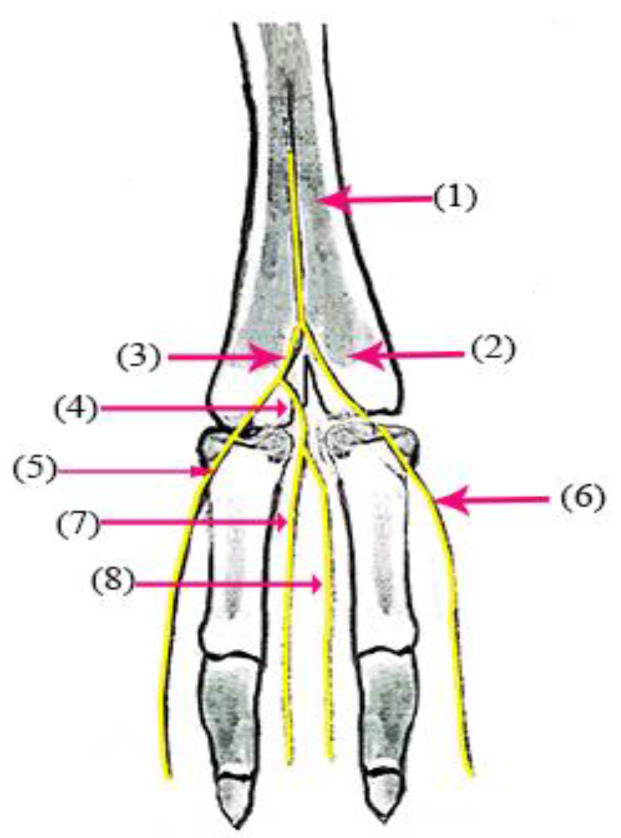
Nerve supply of the dorsal surface of the distal hindlimb in camels: (1) superficial fibular nerve; (2) common dorsal nerve of the IV digit; (3) common dorsal nerve of the III digit; (4) axial dorsal proper nerve of the III digit; (5) abaxial dorsal proper nerve of the III digit; (6) abaxial dorsal proper nerve of the IV digit; (7) axial dorsal proper nerve of the III digit; (8) axial dorsal proper nerve of the IV digit.

**Figure 4:**
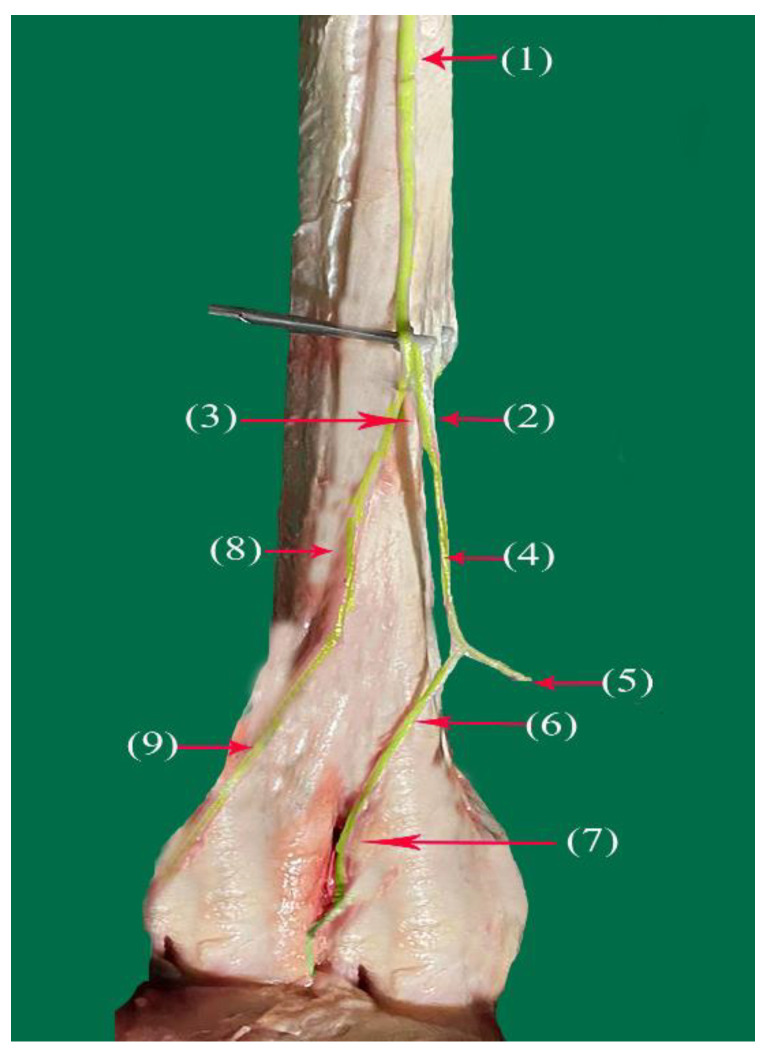
Nerve supply of the plantar surface of the distal hindlimb in camels: (1) tibial nerve; (2) medial plantar nerve of the III digit; (3) lateral plantar nerve of the IV digit; (4) common plantar digital nerve of the III digit (continuation of medial plantar nerve); (5) abaxial plantar proper digital III nerve; (6) interdigital branch of lateral plantar nerve; (7) axial plantar proper digital III nerve; (8) common plantar digital nerve of the IV digit (continuation of lateral plantar nerve); (9) abaxial plantar proper digital IV nerve.

The superficial fibular nerve (Figure 1(1), Figure 2(1) and Figure 3(1)) originates from the division of the common fibular nerve at the condyles of the tibia bone, continues along the dorsal aspect of the metatarsal bones III and IV, and splits into the third and fourth common dorsal digital III and IV nerves at the distal third of the metatarsal bones. (*Nn.dorsalis digitalis communis III and IV*) (Figure 1(2), Figure 2(2) and Figure 3(7)), whereas the medial plantar nerve (*N. medialis plantaris III*) (Figure 4(2)) runs distally.

**Figure 5:**
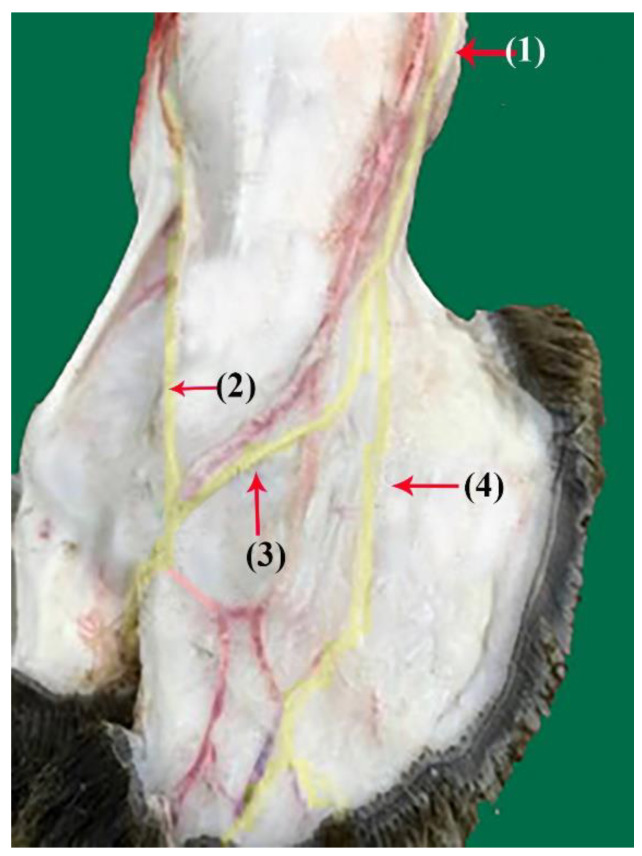
Nerve supply of the dorsal and plantar surfaces of the phalanx of the hindlimb in camels: (1) lateral plantar nerve (common plantar digital nerve of the IV digit); (2) axial dorsal proper digital IV nerve; (3) abaxial dorsal proper digital IV nerve; (4) abaxial plantar proper digital IV nerve.

The common dorsal digital IV descends distally along the metatarsal IV, after which it passes laterally to the long digital extensor to supply the long extensor tendons, then continues along the abaxial surface of the IV digit as the abaxial dorsal proper digital nerve of the IV digit (*N. digitalis proprius dorsalis IV abaxialis*) (Figure 1(4), Figure 2(4), Figure 3(6), and Figure 5(3)). Where the common dorsal digital III (*N. digitalis dorsalis comminus III*) (Figure 2(2)) descends distally along the metatarsal III, it splits into three branches.

1. The axial dorsal proper digital nerve of the IV digit (*N. digitalis proprius dorsalis IV axialis*) continues along the medial surface of the fourth IV digit (Figure 2(5), Figure 3(8) and Figure 5(2)).
2. The abaxial dorsal proper digital nerve of the III digit (*N. digitalis proprius dorsalis III abaxialis*) continues along the lateral surface of the third III digit (Figure 2(6) and Figure 3(5)).
3. The axial dorsal proper digital nerve of the III digit (*N. digitalis proprius dorsalis III Axialis*) continues along the medial surface of the third III digit (Figure 3(7)).

The dorsal surface of the metatarsus and digits receives numerous twigs of nerve branches from the fibular nerve along its extension to the skin of the dorsal surface of the metatarsus. Additionally, the terminal twigs supply the axial and abaxial dorsal aspect of the third and fourth digits, as well as the interdigital surface of the digits.

### 3.2. Tibial Nerve (N. tibialis)

**Figure 6:**
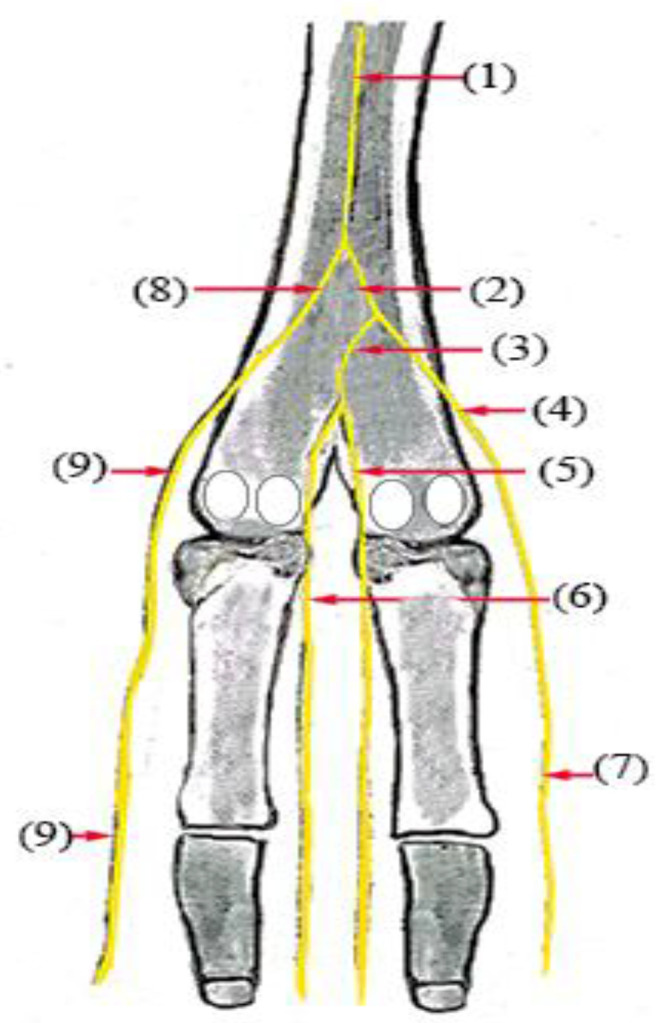
Nerve supply of the plantar surface of the distal hindlimb in camels: (1) tibial nerve; (2) medial plantar nerve of the IV digit; (3) axial plantar proper digital nerve of the III digit; (4) abaxial plantar proper nerve of the III digit; (5) axial plantar proper nerve of the III digit; (6) axial plantar proper nerve of the IV digit; (7) abaxial plantar proper nerve of the III digits; (8) lateral plantar nerve of the IV digit; (9) abaxial dorsal proper digital IV nerve.

The tibial nerve (Figure 3(1) and Figure 6(1)) runs in the plantar aspect of the metatarsal dorsal region to the caudal tibial artery along the medial border of the deep flexor tendon and divides at the proximal third of the metatarsus into two branches, the lateral and medial plantar branches (Nn.lateralis—IV, and medialis plantaris—III, respectively). 

**Figure 7:**
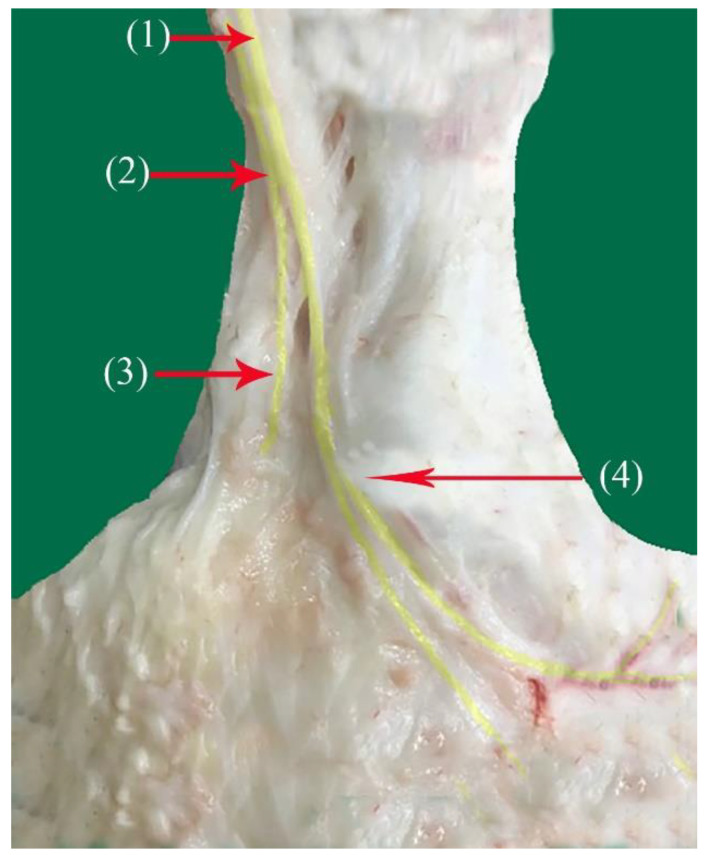
Nerve supply of the plantar surface of the phalanx in camels: (1) medial plantar nerve (common plantar digital nerve of the III digit); (2) interdigit branch of medial plantar nerve; (3) axial plantar proper digital nerve of the IV digit; (4) abaxial plantar proper digital nerve of the III digit.

The medial plantar nerve (*N. medialis plantaris III*) (Figure 4(2), Figure 6(2)) and Figure 7(1)) is longer than the lateral nerve and it runs distally along the medial border of the digital flexor tendon, after which it continues distally along the medio-planter aspect of the suspensory ligament as the common plantar digital nerve of the III digit (*N. digitalis plantaris comminus III*) (Figure 5(4) and Figure 7(1)). It splits into two to three twigs at the suspensory ligament. At the fetlock joint, it divides into two branches, as follows:

1. The abaxial plantar proper digital nerve of the III digit (*N. digitalis plantaris proprius III abaxialis*) (Figure 4(5) and Figure 6(4–7)) runs along the abaxial aspect of the medial digits and supplies the plantar-abaxial aspect of the III digit ending at the foot pad.
2. The axial plantar proper digital nerve of the III digit (*N. digitalis plantaris proporius III axialis*) (Figure 4(7) and Figure 6(3–5)) continues its deep course between the third and fourth digits as the interdigit branch (Figure 6(3–5) and Figure 7(2)), dividing into the following two branches:
  - The axial plantar proper digital nerve of the III digit (*N. digitalis plantaris proporius III axialis*)) (Figure 4(7) and Figure 6(3–5)).
  - The axial plantar proper digital nerve of the IV digit (*N. digitalis plantaris proporius IV abaxialis*) to supply the axial and abaxial aspects of the III and IV digits (Figure 5(4) and Figure 6(6)).

**Figure 8:**
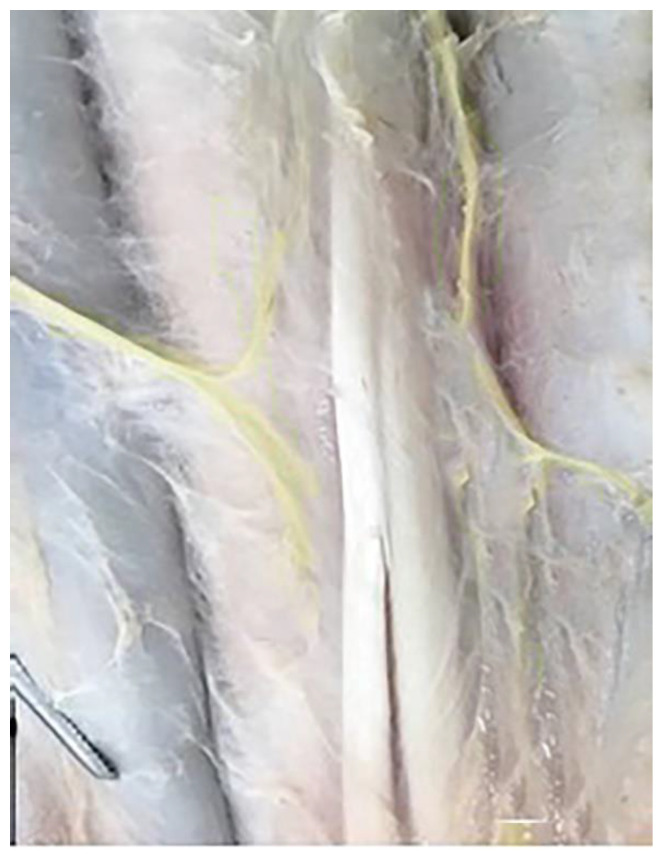
Cutaneous nerves of the medial and lateral surface of the metatarsus region in camels.

**Figure 9:**
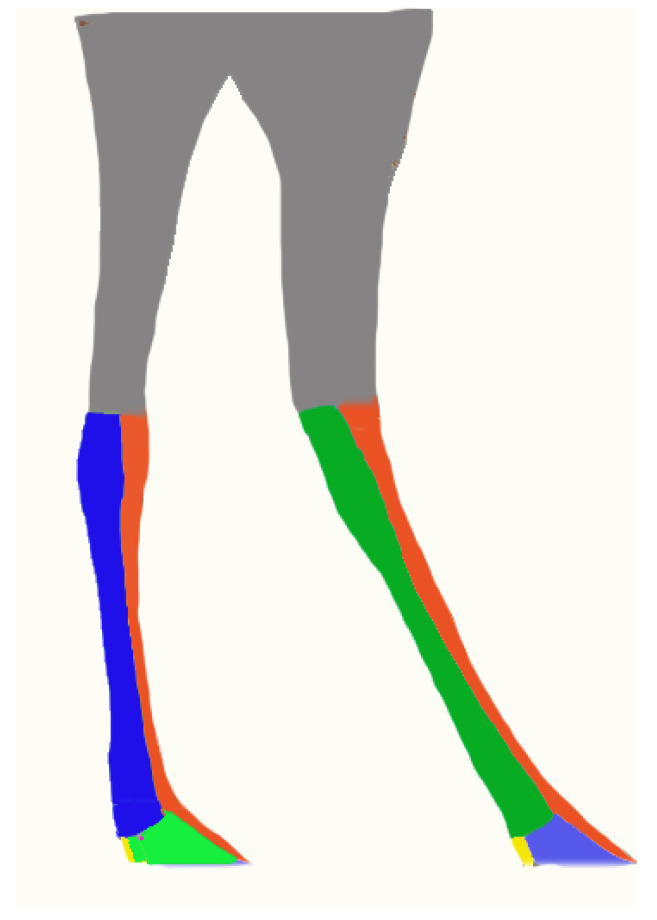
Colors corresponding to the innervation of the cutaneous regions in the distal part of the hindlimbs of camels. 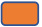 Dorsal surface of the III and IV digits.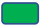 Lateral surface of the metatarsus of the IV digit. 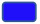 Medial surface of the metatarsus of the III digit. 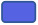 Abaxial surface of the IV digit. 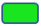 Axial surface of the III digit. 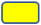 Caudo-plantar surface of the IV digit.

**Figure 10:**
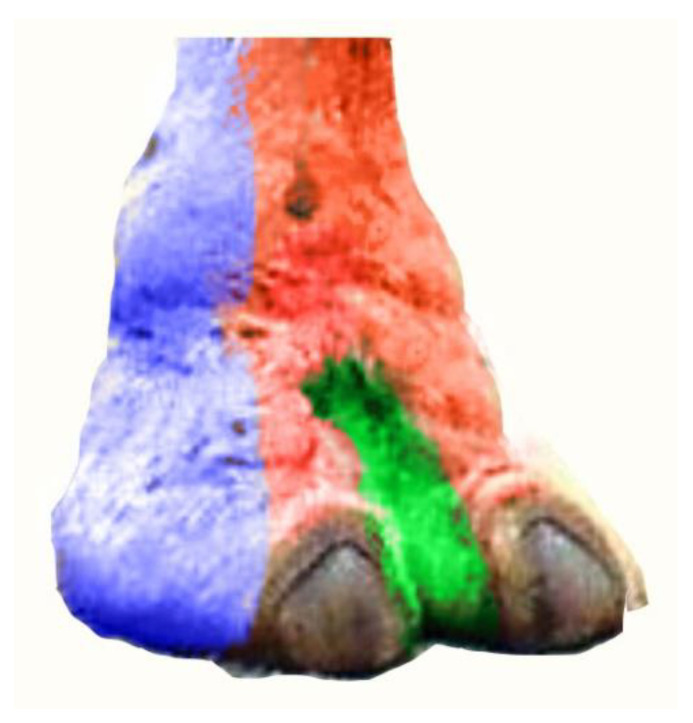
Color corresponding to innervation of the III and IV digits.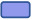 Lateral and latero-plantar aspect innervation by the abaxial lateral tibial nerve of the III and IV digits. 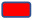 Dorsal aspect innervation by the superficial fibular nerve of the III and IV digits. 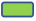 Medial and medio-plantar aspect innervation by the axial medial tibial nerve of the III and IV digits.

The medial plantar nerve of the tibial nerve has numerous branches along its extension to the skin of the medio-plantar surface of the metatarsus (Figure 8). Additionally, the terminal twigs supply the axial and abaxial plantar aspects of the third digit and the abaxial plantar surface of the fourth digit, as well as the skin of the interdigital surface and the abaxial aspect of the skin of the digits (Figure 9 and Figure 10).

### 3.3. The Lateral Plantar Branch (N. lateralis plantaris IV)

The lateral planter branch (Figure 4(3), Figure 5(1) and Figure 6(8)) is thinner than the medial branch and passes on the lateral border of the suspensory ligament. It continues in the latero-plantar aspect of the metatarsus as a common plantar digital nerve of the IV digit (*N. digitalis plantaris communis IV*) (Figure 4(8) and Figure 5(1)) (common plantar digital IV). It descends along the planto-lateral aspect of the metatarsus of the IV digit. Above the fetlock joint, it divides into the following two branches: the medial (interdigital) branch and the lateral branch.

The interdigital branch (*Interdigitalis b*) (Figure 4(6) and Figure 7(3)) continues as an axial plantar proper digital nerve of the IV digit (*N.digitalis plantaris proprius IV axialis*) (Figure 4(5)). It supplies the interdigital surface of the digits and the tendon sheet. It splits into a plantar twig, extending to the distal plantar in the fascia and skin of the interdigit aspect of the fetlock and pastern joint (Figure 10).

The lateral branch (*Lateralis. b*) of the lateral plantar nerve at the fetlock joint continues on the lateral aspect of the IV digit as an abaxial plantar proper digital nerve of the IV digit (*N. digitalis plantaris proprius IV abaxialis*) (Figure 4(9) and Figure 6(9)), crossing along the abaxial aspect of the lateral IV digits and supplying the plantar-abaxial aspect of the IV digit, ending at the foot pad. The lateral plantar nerve (*N lateralis plantaris IV*) supplies the plantar-abaxial aspect of the fourth digit’s skin (Figure 9 and Figure 10). 

The superficial fibular nerve contributes to the nerve supply of the skin of the dorsal surface of the metatarsus region. In addition, the terminal branches of the superficial fibular nerve supply the dorsal surface of the axial and abaxial aspects of the third and fourth digits (Figure 9 and Figure 10).

The numerous twigs of nerve branches of the caudal sural cutaneous nerve from the tibial nerve involve the lateral and plantar aspects of the leg to the metatarsus and digits innervation along its extension to the skin of the plantar surface of the metatarsus (Figure 6). In addition, the terminal twigs supply the axial and abaxial plantar aspects of the third and fourth digits, as well as the interdigital surface of the digits (Figure 9 and Figure 10). The numerous twigs of nerve branches contribute to the saphenous nerve in the dorsomedial aspect of the leg to the metatarsus and digit innervation, which also contributes with branches to the III and IV digits, along with the I and II digits.

## 4. Discussion

The nerves that supply the innervation of the leg and pes of camels are the saphenous, superficial fibular, tibial, caudal cutaneous sural and lateral cutaneous sural nerves, which agrees with records in domestic animals including camels [12,13,14,15,21,22]. Some authors identified the superficial fibular nerve and the dorsal pedal III and IV nerves in this system [12,13].

The anatomy of hindlimb nerves that reach the leg is not described in full detail in many species, since authors have rarely addressed the distribution of nerves distally to the stifle joint. This area requires greater accuracy due to the dissection process of the hindlimb. However, it was described in detail only in the giant anteater [3].

In the present study, in the distal third part of the metatarsal bones, the superficial fibular nerve was found to divide into two branches, namely the common dorsal digital III and IV nerves. Our results disagree with what was previously reported in camels and cattle [15,17]. For instance, the position of the split into the common dorsal digital III and IV nerves was in the middle third of the metatarsus region. On the other hand, the superficial fibular nerve divides into the common dorsal nerves of the III and IV digits [17]. The lateral sural cutaneous nerve was a branch of the common fibular nerve. Our results are in agreement with those for domestic animals by [13,15,16,17] and in camels by [23].

Dyce et al. (2010) recorded that the superficial fibular nerve divides into the common dorsal nerves of the III and II digits [14]. These findings are in disagreement with our results, which revealed that the superficial fibular nerve divides into the common dorsal nerves of the III and IV digits. Cruze et al. 2014 recorded that the dorsal metatarsal branches of the deep fibular nerve are absent, where the saphenous nerve is involved in the digital innervation with branches to digits III and IV, in addition to digits I and II in the giant anteater [3].

The common dorsal digital IV nerve continues as the abaxial dorsal proper digital nerve of the IV digit. These results are consistent with the previously reported locations in camels [24,25], in cattle [17], and in buffalo [25].

The common dorsal digital III nerve in our work has three branches, including the axial dorsal proper digital nerve of the IV digit, the axial dorsal proper digital nerve of the III digit, and the abaxial dorsal proper digital nerve of the III digit. These results disagree with what was reported [24] in camels and [25] in buffalo, which suggests that the third dorsal pedal III nerve at the fetlock joint divides into the third and fourth common dorsal pedal digital nerve, where the middle branch divides between the digits into the axial and axial dorsal digit pedal III.

The dorsal aspect of the pes was dorsally innervated by the abaxial dorsal proper digital nerve of the IV digit from the common dorsal digital IV nerve and the axial and abaxial dorsal proper digital nerves of the III digit from the common dorsal digital III nerve of the superficial fibular nerve. The relevant findings disagree with [14,23,24] in camels, [17] in cattle, [25] and in buffalo, which recorded that the pes received the nerve supply from dorsal common digital nerves II–IV from the superficial fibular nerve and in the interdigital region by the dorsal metatarsal nerve III.

In domestic animals, the foot structures’ sensitive innervation was investigated [13]. It is mediated by the combination of dorsal metatarsal structures originating from the deep fibular nerve with the dorsal digital branch, which in turn originates from the superficial fibular nerve. However, the deep fibular nerve does not have dorsal metatarsal branches in camels.

In the dorsal section of the hindlimb, the superficial fibular nerve continues to be the dorsal common digital nerve. The dorsal common digital nerve splits into the dorsal proper digital nerves, which feed the axial and abaxial surfaces of the corresponding digits [26,27,28,29]. In pigs, the saphenous nerve divides into lateral and medial branches at the distal third of the leg, which is similar to our results. The medial ramus of the superficial fibular nerve joins the lateral ramus of the saphenous, continuing as the dorsal common digital nerve II, whereas the medial ramus descends to become the dorsal medial digital nerve II [30]. These findings are in disagreement with our results that revealed that the superficial fibular nerve divides the common dorsal nerve of the III and IV digits. Our results, however, are similar to those reported in dogs, cats and in African lions.

The tibial nerve’s division into medial and lateral plantar nerves confirms the pattern described in goats [12], domestic animals [13], camels [15,23,24] and other species [5,6,14,19,30]. However, the correlation between the formation of the digital nerves and the number of digits plays a critical role in the distal distribution of this nerve, as reported in carnivores, which have a larger number of digits.

The current study revealed that the tibial nerve divided at the proximal third of the metatarsus into two branches, namely the lateral and medial plantar branches. This result is inconsistent in camels, as in other studies, the division of the tibia nerve was observed at the tarsus [14], while it was observed at the distal end of the crus in bovines [17]. The starting location of the caudal tibia cutaneous nerve varies among different species. It arises from the tibial nerve in dogs and bovines [30].

The medial plantar branch continues as a common plantar digital nerve of the III digit. Our findings disagree with those of other studies on camels, which reported that the medial plantar nerve continues as the common plantar digital nerve of the II digit [23].

Our results confirm that the medial plantar branch continues as the abaxial plantar proper digital nerve of digit IV in domestic animals [13], in bovines [17], and in camels [15,23,24].

The medial plantar branch divides into the axial plantar proper digital nerve of the III digit and the abaxial plantar proper digital nerve of the III digit. Similar findings were reported in previous studies in domestic animals [12,13]. In contrast, the medial plantar nerve splits into the axial plantar digital V and the abaxial plantar digital V [16]. The abaxial plantar proper digital nerve of the III digit is one terminal branch of the common plantar digital nerve of the III digit in interdigital space [23].

Smuts et al. (1986) recorded that the medial plantar nerve divides into common plantar digital nerves of the II and III digits [15]. In contrast, other studies concluded that the abaxial plantar proper digital nerve of the III digit was the inner division of the common plantar nerve of the II digit. However, the axial plantar proper digital nerve of the III digit and the axial plantar proper digital nerve of the IV digit were due to the division of a common plantar nerve of the III digit in interdigital space [23,24].

In this study, the lateral plantar nerve divides into the following two branches: the medial (interdigital) branch and the lateral branch. The lateral branch continues as the abaxial plantar proper digital nerve IV of the digit and supplies the plantar-abaxial aspect of the IV digit. These findings are in agreement with the results of other studies in camels [14,23,24], in bovines [17], and in domestic animals [13,18,25].

Our results show the medial plantar digital nerve bifurcates from the tibial nerve at the tarsus level. They both continue together distally to the middle of the metatarsal bones. Then, they divide into the plantar common digital nerves II–IV. After that, they continue medially, becoming the plantar proper digital II abaxial nerve. Our results are similar to the findings reported in dogs [29,31] and in African lions [28]. In contrast, another study reported that the lateral plantar nerve comes from the distal deep plantar arch, is very short, and divides into axial plantar digital nerve V and abaxial plantar digital nerve IV, which are distributed as the corresponding structures of digit II, as well as splitting into a deep branch to the interossei III and IV in bovines [16].

The saphenous nerve distribution on the cranial and medial surface of the camel leg was similar to that described in bovines [9], camels [15], horses [18], and domestic animals [32,33,34]. In conclusion, the nerve supply of the distal portion of the hindlimb is similar to what was described in other ruminants [17].

Finally, these results show that the nerve distribution of the distal parts of the hindlimbs are of great importance, because they will help in the determination of the regional anesthesia of the different nerves in the distal hindlimb, especially in the treatment of tendonitis, osteoarthritis and sesamoiditis [35,36]. On the other hand, injuries to these nerves lead to a variety of deficits, including a loss of sensitivity, physical action, or both [37].

## 5. Conclusions

The findings show the anatomical structure of the nerve supply of the distal hindlimb in dromedary camels. We showed the nerve blocks and their distribution within this region, including the skin, tendons and joints. The findings will assist in successful anesthesia and surgery in this region. 

## Data Availability

Pictures and other relevant information for this research are available upon request from the corresponding author, shnbry@qu.edu.sa.
